# Plasmodium falciparum infection status in children less than 10 years old under seasonal malaria chemoprevention and risk of clinical malaria in the Koulikoro health district, Mali

**DOI:** 10.21203/rs.3.rs-4613312/v1

**Published:** 2024-07-18

**Authors:** Daouda Sanogo, Mahamoudou Toure, Moussa Keita, Fousseyni Kane, Soumba Keita, Ibrahim Sanogo, Sory Ibrahim Diawara, Hamady Coulibaly, Sidibé M’Baye Thiam, Mahamadou Diakite, Nafomon Sogoba, Seydou Doumbia

**Affiliations:** University of Sciences, Techniques and Technologies of Bamako; University of Sciences, Techniques and Technologies of Bamako; University of Sciences, Techniques and Technologies of Bamako; University of Sciences, Techniques and Technologies of Bamako; University of Sciences, Techniques and Technologies of Bamako; University of Sciences, Techniques and Technologies of Bamako; University of Sciences, Techniques and Technologies of Bamako; University of Sciences, Techniques and Technologies of Bamako; University of Sciences, Techniques and Technologies of Bamako; University of Sciences, Techniques and Technologies of Bamako; University of Sciences, Techniques and Technologies of Bamako; University of Sciences, Techniques and Technologies of Bamako

## Abstract

**Introduction:**

Seasonal malaria chemoprevention (SMC) with Sulfadoxine pyrimethamine plus amodiaquine (SP + AQ) consist of a monthly administration of therapeutic dose to children under five years of age during the high risk of malaria in area where malaria is highly seasonal. According to SMC recommendation, both non-infected and asymptomatic *Plasmodium falciparum* infected children will receive similar treatment. The gap in our knowledge is how the effect of asymptomatic infection on the efficacy of SMC in preventing clinical malaria over a four-week period. Thus, this study aimed to assess the risk of clinical malaria and its association with children’s infection status when SMC treatment is given.

**Methodology:**

The study was carried out in the Koulikoro health district in Mali and concerned children under 10 years of age. A total of 726 and 1452 children were randomly selected and followed over the SMC campaign in the years 2019 and 2020 respectively. Prevalence of asymptomatic *P. falciparum* infection was determined each round by microscopy before SMC drugs intake. Children were passively followed over a four-week period to determine incidence of clinical malaria. R-Studio software was used for analysis. The risk of clinical malaria by infection status was estimated using a logistic regression. A Kaplan-Meier curve was used to determine the survival time between infected and uninfected children. The Pearson Chi-square test was used to compare proportions with the significant level at p< 0.05.

**Results:**

The average prevalence of asymptomatic infection was 11.0% both years, and it was higher among children aged 5 to 9 years old in 2019 (p<0.001) and 2020 (p=0.016). The risk of clinical malaria was significantly higher among asymptomatic infected children 2019: (RR=3.05, CI [2.04–4.72]) and 2020 (RR=1.43, CI [1.04–1.97]) transmission seasons. Likewise, the time of the first malaria occurrence was statistically lower among infected children regardless the year (p<0.001 in 2019 and p=0.01 in 2020).

**Conclusion:**

Results show a high risk of clinical malaria in asymptomatic infected children during SMC delivery. Screening for *P. falciparum* infection before the SMC treatment could significantly enhance the impact of the strategy on malaria morbidity in endemic areas.

## Background

Seasonal malaria chemoprevention (SMC) has been recommended by the World Health Organization (WHO) for malaria prevention in children under five years in Sub-Saharan African countries where the transmission is highly seasonal since 2012. It involves the regular administration of antimalarial drugs on a monthly basis for a duration of three days, namely during periods characterized by elevated malaria transmission rates, which typically occur over a span of three to five months annually[1, 2]. Progressively, SMC has been deployed in specific regions of Mali as pilot studies from 2012 to 2015, then the strategy becomes in 2016 a countrywide malaria prevention tools for children less than five years. Several studies have shown that SMC significantly reduces burden by reducing malaria-related morbidity, mortality and malaria anemia [1–5]. Despite the proven effectiveness of the strategy, malaria remains the most common and deadly disease in Mali with 3,204,275 confirmed cases and fatality rate at 1.4%_0_ in 2022 according to the National Malaria Control Report (NMCP) [6]. Asymptomatic carriage of parasites consists in the absence of clinical manifestations despite the presence of parasites in the blood. These individuals are very important for transmission because they constitute a reservoir of parasites [7–11].

As per WHO recommendations, only symptomatic children must be tested for malaria before the intake of SP + AQ while malaria RDT test is not required in the absence of symptom. Thus, in malaria endemic area, a significant proportion of asymptomatic infection may receive SMC without knowing their infection status. Since the implementation of SMC as community intervention in malaria endemic region of sub-Saharan Africa, fewer studies have assessed the possible impact of asymptomatic infection on the success of the strategy to protect against clinical malaria among eligible children. that SMC could have on the success of taking drugs in asymptomatic infected children. A risk assessment of the incidence of clinical malaria with respect to infection status at the time treatment is given could help explain the occurrence of the disease among children within four weeks after receiving SMC treatment. This study conducted during the SMC campaign will try to estimate the risk of presenting with malaria symptoms plus a positive RDT within four weeks after completing SMC treatment among eligible children living in Koulikoro health district of Mali. Result could inform on how giving SMC only to non-infected children could increase the likelihood of not having clinical malaria among treated population in endemic area.

## Methods

### Study sites.

**Figure 1:**
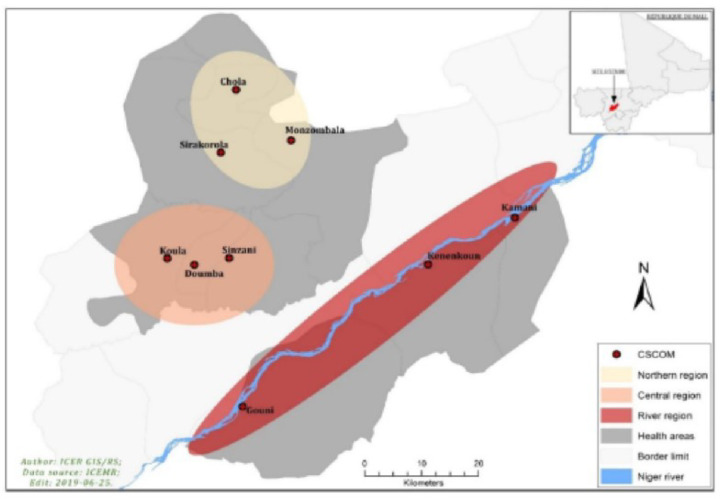
Study site

The study was carried out in the health district of Koulikoro located in the tropical zone of Mali, at 60 kilometers from the capital Bamako, Within the district there is different ecological patterns leading to different length of the malaria transmission season (from 4 to 5 months a year). A total of nine (9) villages, both having a community health center and representing both ecological patterns. Sirakorola, Chola, Monzombala were in the dry area with a short transmission season (3 months), Doumba, Sinzani, Koula with a transmission season over four months, Gouni, Kenenkou and Kamani, located along the river; with a transmission season lasting for five months (Fig. 1).

### Study population.

The target population was those eligible for SMC (children aged 3 months to less than 10 years). After a census enumeration of each village, the total population size of the 9 villages was approximately 27,867 with 6,326 and 6,638 children eligible for SMC in 2019 and 2020 respectively.

### Sampling

**Figure 2:**
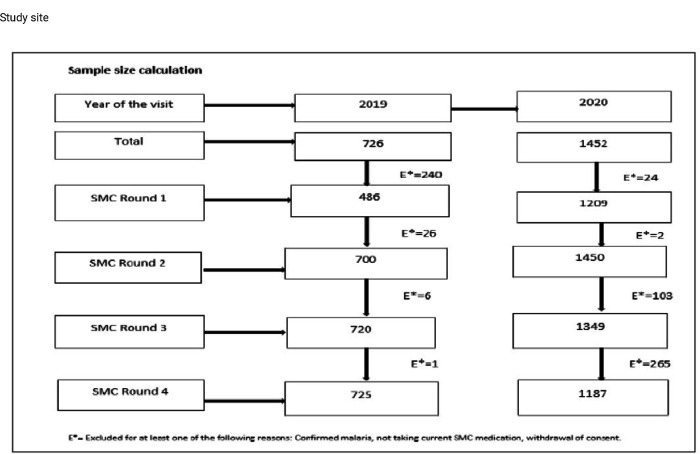
Sample size calculation

Prior to the SMC season, within all villages, parents, or guardians of children under 10 years of age eligible for SMC were asked for voluntary consent and only upon completing a signed consent form, children were enrolled and given a unique identifying number for the study. A sub sample of 726 and 1452 children enrolled respectively in 2019 and 2020 was then chosen randomly per village to be tested for asymptomatic *P. falciparum* infection by microscopy before the SMC drug administration. The Fig. 2 (Fig. 2) shows the sample size estimation per year and per month.

The sample size was 726 children in 2019 and for more statistical power, this size was double in year 2020 for a total of 1452 children. Monthly sample size was calculated based on the seasonal variation of asymptomatic malaria prevalence in the study area.

### Data collection and collection tools

Before each SMC delivery, parasite assessment was done for each participant and the follow up consisted of a passive case detection of clinical malaria at the community health center. Each year, the study was conducted from July (first SMC round) to November (a month after the last SMC round) Sociodemographic, clinical symptoms as well as malaria RDT test and smear were done at each visit. Electronic data capture was used to collect data through the Redcap platform and synchronized daily.

### Statistical analysis

Redcap data were exported as an Excel file for further analysis in the statistical program R version 4.2. To compare percentages, we used the Chi-squared test, and to analyze risk across groups for infection, we utilized logistic regression. Statistical significance was assumed when the p-value was less than 0.05. The Kaplan Meier method was then used to estimate how long it would be before the first clinical malaria episode occurred after SMC therapy.

## Results

### Descriptive of study population.

On average, children under five years old accounted for 52.9% (1394/2631), while those aged 5–9 years old were 47.1% (1237/2631) in 2019. These proportions were 48.0% (2494/5195) and 52.0% (2701/5195) for children aged 3–59 months and 5–9 years respectively in 2020 (Table 1).

### The prevalence of Plasmodium falciparum asymptomatic infection by month, age group and year.

**Figure 3:**
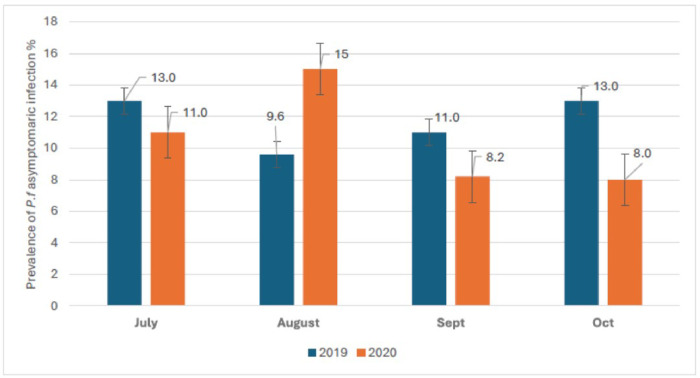
Prevalence of asymptomatic *P. falciparum*carriage by SMC round by year.

**Figure 4:**
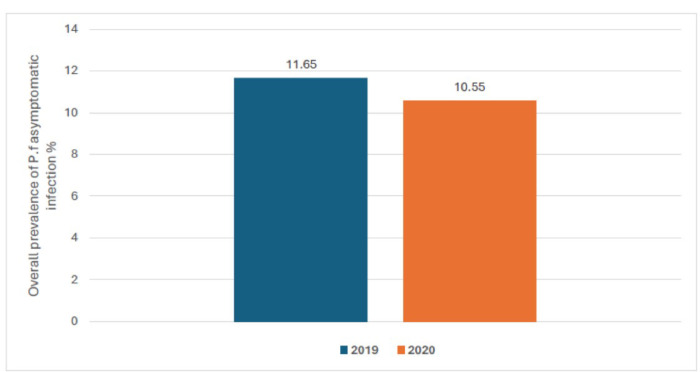
overall prevalence of asymptomatic *P. falciparum* carriage by year

**Figure 5:**
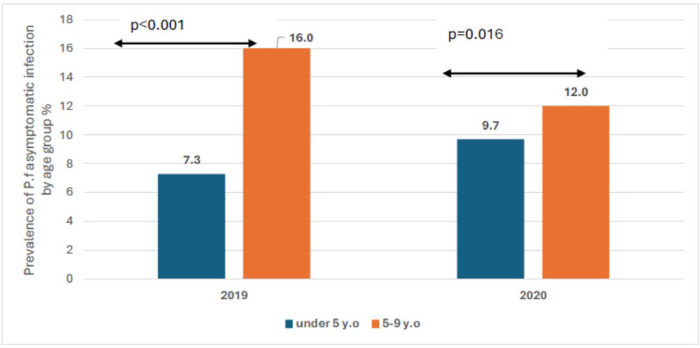
overall age specific prevalence of asymptomatic *P. falciparum* carriage per year

The prevalence of *Plasmodium falciparum* asymptomatic infection was respectively 13.0%, 9.6%, 11.0% and 13.0% in July, august, September and October of 2019. It was 11.0%, 15.0%, 8.2%, and 8.0% respectively in July, august, September and October of 2020 (Fig. 3). The overall prevalence of malaria infection during the season was 11.65% in 2019 and 10.55% in 2020 (Fig. 4). Regardless of the year, the age-specific prevalence shows significantly high prevalence of asymptomatic infection among older children aged 5 to 9 years old compared to children under five years old. We observed respectively 7.3% vs. 16.0% in year 2019 (p < 0.001) and 9.7% vs. 12.0% in year 2020 (p = 0.016) (Fig. 5).

### The overall incidence of clinical malaria according to asymptomatic Plasmodium falciparum carriage prior to SMC medications.

The average cumulative incidence of clinical malaria was 23.58% among asymptomatic children compared to 7.47% among healthy children in 2019 (RR = 3.16, 95% CI [2.49–40]; p < 0.001). In 2020, the cumulative clinical incidence of malaria was 3.60% among asymptomatic children and 5.19% among healthy children (RR = 1.45; 95% CI [1.12–2.16]; p = 0.047) (Table 2).

### The likelihood of becoming clinically symptomatic over time by asymptomatic statute before SMC.

**Figure 6:**
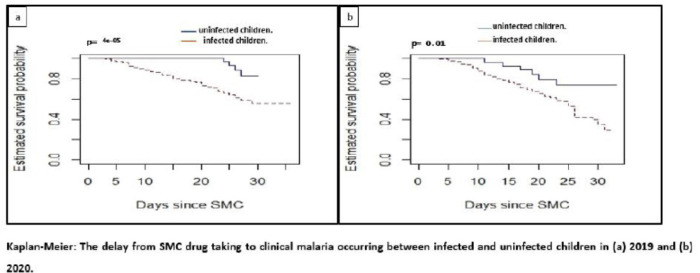
Kaplan-Meier: The delay from SMC drug taking to clinical malaria occurring between infected and uninfected children in (a) 2019 and (b) 2020.

Survival curves between the two groups based on infection status prior to the first SMC treatment are presented in Figs. 6 and 7. Approximately 50.0% (2019) and 75.0% (2020) of infected children show up at the clinic within four weeks with clinical malaria after receiving SMC treatment. In both years, children who were smear negative had a higher survival probability compared to those who were smear positive. Children who have taken the SMC and are infected are more likely to have clinical malaria than those who were not infected when they took the SMC (p < 0.001 in 2019 and p = 0.01 in 2020).

## Discussions

### Methodological approaches

SMC remains an important intervention by reducing malaria-related morbidity and mortality among target population in seasonal transmission areas. Asymptomatic *P. falciparum* carriage play important role in the maintain of malaria transmission and thus, could slow down the progress toward elimination [11–16]. As SMC strategy did not recommend a test-to-treat approach for all eligible children, those with no malaria like symptom at the administration of SMC may receive SP + AQ while carrying the parasite sometimes at low density in their blood. Our study explored the risk of clinical malaria after SMC treatment with respect to infection status among children less than 10 years old during two malaria transmission seasons.

### The prevalence of asymptomatic malaria parasitemia

Our data shows that while SMC is widely implemented with significantly high coverage rates reported in countries like Mali, a non-negligible proportion of children receiving SMC treatment (about 11.0%) are already carrying *P. falciparum* without any symptoms. Studies have shown that in malaria-endemic countries, there is a high prevalence of asymptomatic carriage of *P. falciparum* [9, 17–21] and that this parasitemia remains detectable in some children despite the administration of SP plus AQ during SMC [22].

Prevalence was statistically higher among older children compared to those less than 5 years old as reported elsewhere and defined as an age-shift in malaria prevalence and incidence in sub–Saharan Africa with older children being more at risk. Similar observations were made in Gamby by Ahmad and al. [20] and in Mali by Tran TM. and al [23], Toure M. and al.[24] and Coulibaly D. and al. [25].

### Clinical malaria incidence after taking seasonal malaria chemoprevention drugs and asymptomatic malaria parasitemia carriage before

Asymptomatic infection prior to SMC delivery was significantly associated with high risk of clinical malaria over the next four weeks as shown here. While SP + AQ remain effective to prevent malaria in Mali, they are not recommended as first or second line by the National Malaria Control Program (NMCP). Thus, one can state that this combination by failing to clear asymptomatic infection reservoir, will not prevent from developing clinical malaria within the next four weeks. The survival analysis shows a short time between SMC treatment and symptoms appearance among children infected while receiving SP + AQ compared to those not infected. It is known that one of the principles of SMC is to maintain an optimal concentration of SP and AQ over four weeks leading to prevent any new infection during this period [2, 26], however the efficacy of SP + AQ as a therapy in most endemic countries for *Plasmodium falciparum* malaria has not been well studied since the implementation of SMC, as is the case with its widely recognized effectiveness as a chemoprevention agent [27–29]. Furthermore, malaria parasite sensitivity to SMC drugs as well as the parasite load in blood at the time of SMC treatment could impact the drugs metabolite and the pharmacokinetics leading to short protection[22]. Parasite pressure on SMC drugs is also known to reduce its concentration on plasma of asymptomatic infected children which can lead to a reduce protection against clinical malaria by reducing the strength of prophylaxis inducing by par SMC drugs[22, 30, 31].

## Conclusion

Asymptomatic infection remains significant among SMC eligible children in malaria endemic area and risk of clinical malaria is significantly high among infected children. Mass drug administration of antimalarial treatment aiming to clear parasite reservoir before the first round of SMC campaign in this area could significantly reduce the disease burden during the transmission season.

## Figures and Tables

**Table 1:** Socio-demographic characteristics

Characteristic	2019					2020				
	July = 486	August, n = 700	September, n = 720	October, n = 725	Overall, N = 2,631	July, n = 1.209	August, n = 1.450	September, n = 1.349	October, n = 1.187	Overall, N = 5,195
	n (%)	n (%)	n (%)	n (%)	n(%)	n (%)	n (%)	n(%)	n (%)	n (%)
**GENDER**										
**Male**	253 (52.1)	371 (53.0)	396 (55.0)	377 (52.0)	1,394 (52.9)	677 (55.9)	754 (52.0)	728 (53.9)	617 (51.9)	2,753 (52.9)
**Female**	233 (47.9)	329 (47.0)	324 (45.0)	348 (48.0)	1,237 (47.1)	532 (44.1)	696 (48.0)	621 (46.1)	570 (48.1)	2,442 (47.1)
**Age**										
**Under 5 y.o**	252 (51.85)	399 (57.0)	382 (53.0)	377 (52.0)	1,394 (52.9)	556 (45.90)	696(48.0)	661 (48.9)	605(50.9)	2,494(48.0)
**5–9 y.o**	234 (48.15)	301 (43.0)	338 (47.0)	348 (48.0)	1,237 (47.1)	653 (54.1)	754 (52.0)	688 (51.1)	582 (49.1)	2,701 (52.0)

**Table 2:** The overall incidence of clinical malaria according to asymptomatic *Plasmodium falciparum* carriage prior to SMC medications.

Year of visit	*P. falciparum* Infection status before SMC by microscopy	Cumulative malaria Incidence per 100 during SMC season	RR 95% [IC]	P
**2019**	Negative	7.47		<0.001
	Positive	23.58	3.16[2.49–40]	
**2020**	Negative	3.60		0.047
	Positive	5.19	1.45[1.12–2.16]	
**List of fi gures**		

## Abbreviations

SMC: Seasonal Malaria Chemoprevention
SP+AQ: Sulfadoxine-Pyrimethamine+ Amodiaquin
RDT: Rapid Diagnostic Test
CI: Confidence Interval
*P. falciparum*: Plasmodium falciparum
WHO: World Health Organization
RR: Relative Risk
NMCP: National Malaria Control Program
